# Development and validation of the Transgender Adolescent Stress Survey–Minority Stress (TASS-MS)

**DOI:** 10.3389/fpsyg.2024.1448693

**Published:** 2024-12-04

**Authors:** Jeremy T. Goldbach, Sheree M. Schrager, Jules K. Wood, Rory P. O’Brien, Shannon L. Dunlap, Harmony Rhoades

**Affiliations:** ^1^Sexuality, Health and Gender Center, George Warren Brown School of Social Work, Washington University in St Louis, Saint Louis, MO, United States; ^2^Office of Graduate Studies and Research, California State University-Dominguez Hills, Carson, CA, United States; ^3^School of Social Work, University of Michigan, Ann Arbor, MI, United States; ^4^Luskin School of Public Affairs, University of California, Los Angeles, Los Angeles, CA, United States

**Keywords:** transgender adolescents, non-binary adolescents, minority stress, behavioral health, measure development (psychometrics)

## Abstract

**Objective:**

This study aimed to create and validate a novel measure of gender-related minority stress in transgender and non-binary adolescents (TNBA). TNBA face higher risks of varied behavioral health concerns compared to their cisgender peers, a disparity often attributed to the presence of minority stress due to discrimination. To date, no comprehensive measures of gender-related minority stress exist for use with TNBA.

**Method:**

The present study recruited a U.S. national sample (*N* = 444, aged 12–17; 65.5% White, 9.5% Black, 9.5% Latine, 15.5% other ethnicity; 34.7% transmasculine, 17.3% transfeminine, 38.3% non-binary, 9.5% agender) of TNBA. An initial item pool was developed from life history calendars, a modified Delphi process, and cognitive interviews with TNBA. Analytic methods including principal components analysis, item response theory, measurement invariance testing, and reliability analyses were conducted to establish the final scale. Concurrent validity was established across behavioral outcomes (mental health, suicidal thoughts and behavior, substance use), and convergent and divergent validity compared the Transgender Adolescent Stress Survey–Minority Stress (TASS-MS) to existing measures of gender-related minority stress.

**Results:**

The TASS-MS and its subscales (disaffirmation, visibility and internalized transnegativity, family) were significantly associated with anxiety and depressive symptoms, PTSD symptoms, suicidal behaviors, non-suicidal self-injury, marijuana, and prescription drug use. The TASS-MS was moderately and weakly correlated with convergent and divergent measures, respectively, indicating specificity to minority stress.

**Conclusion:**

The TASS-MS is a reliable and valid measure for future research with TNBA. It is inclusive and usable by all gender minority adolescents, uses a standard simple scoring system, and assesses adolescent-specific stressors.

## 1 Introduction

Evidence from both national and community-based studies shows that transgender and non-binary adolescents (TNBA; adolescents whose gender identity is different than their sex assigned at birth) are at significantly higher risk of behavioral health concerns such as substance use, anxiety, depression, self-harm, and suicidality compared to their cisgender peers (Centers for Disease Control and Prevention, 2017; Peterson et al., 2017; Toomey et al., 2018). TNBA report strikingly high prevalence of lifetime suicide attempts, with 30–51% having ever attempted suicide compared with 7% among cisgender heterosexual adolescents (Centers for Disease Control and Prevention, 2017; Peterson et al., 2017; Toomey et al., 2018).

Although researchers have historically combined TNBA with cisgender sexual minorities to create a single group (i.e., LGBT), TNBA consistently report greater disparities than their cisgender sexual minority adolescent (SMA; e.g., lesbian, gay, bisexual) peers. For example, although 23% of sexual minorities nationally report a lifetime suicide attempt (Goldbach et al., 2015), this rate is 32.3% among TNBA (Kuper et al., 2018). These differences are evident in symptoms of anxiety (18.7% vs. 33%, respectively; Bockting et al., 2013, 2016; Bostwick et al., 2010), depression (18% vs. 44%; Bockting et al., 2013; Russell and Fish, 2016), substance misuse (30% vs. 49%; Reisner et al., 2015; Watson et al., 2018), PTSD symptoms (Russell and Fish, 2016; Wharton, 2007), and suicide attempt (Bockting et al., 2016; Russell et al., 2011). Given these disparities, we contend that the factors that drive TNBA behavioral health are (at least in some ways) distinct from those that drive SMA health.

Health disparities among both sexual and gender minority people are often attributed to the presence of minority stress. Minority stress theory has been endorsed by the Centers for Disease Control and Prevention (2023), National Academy of Medicine (2015), and Healthy People 2030 (Office of Disease Prevention and Health Promotion, n.d.). This theory suggests that a pervasive anti-LGBT culture predisposes LGBT people to excess stress, as compared with cisgender heterosexual people. This stress, in turn, leads to adverse health outcomes and drives health disparities (Clark et al., 2018; Eisenberg et al., 2017; Meyer, 2003; Perez-Brumer et al., 2017).

Minority stress theory was initially developed as a framework for understanding behavioral health among sexual minority adults; however, prior research has described several minority stressors that are unique to TNBA (Testa et al., 2015) and the developmental period of adolescence (Goldbach and Gibbs, 2017). The adaptation of the minority stress model to gender minority experiences highlighted additional stressors that have been proposed to explain differences in TNBA and cis-SMA health outcomes. Such differences include distal stressors around gender identity disclosure with family and peers (Bockting et al., 2016; Nuttbrock et al., 2010; Olson et al., 2011), in-school victimization (bullying) by both students and faculty members (Perez-Brumer et al., 2017; Reisner et al., 2015; Yunger et al., 2004), experiences of childhood violence (Centers for Disease Control and Prevention, 2023; Deogracias et al., 2007; National Academy of Medicine, 2015), and loss of housing attributed to disclosure to parents (Kelleher, 2009; Russell and Fish, 2016; Savin-Williams, 2001). It also includes proximal stressors of well-being, including self-concept and internalized distress (Grossman et al., 2021; Meadow, 2012; Simons et al., 2013), identity concealment, and anticipation of rejection (Bockting et al., 2016), which can lead to delays in access to care. For example, to access medical care (including assessment, puberty blockers, hormones, or surgery), TNBA often must rely on parental consent (Clatts et al., 2005). As such, parents are often the gatekeepers to TNBA’s access to physical and psychological gender affirmation and care. Similarly, lack of knowledge and support by providers in health care settings (especially for younger adolescent patients), an increasing number of statewide bans on gender-affirming care (Conron et al., 2022), and the use of mental health therapists as gatekeepers to determine if someone is “trans enough” to access hormone therapy appear to be common (Schulz, 2018).

Given the distinct experiences of TNBA from their cisgender SMA counterparts, the lack of comprehensive measures of gender-related minority stress for use with TNBA is a notable gap in the scientific literature. Indeed, a recent review of eight measures of transgender stress by Shulman et al. (2017) found that most measures lack test–retest validity, many assess a limited range of issues regarding identity congruence or community belongingness (Johns et al., 2017), and several make the incorrect assumption that all transgender persons have the same or similar end goals (e.g., medical transition; Sandler et al., 1997).

To our knowledge, only one measure has focused on measuring a diverse set of minority stress domains for transgender persons. In their Gender Minority Stress and Resilience Measure (GMSR), Testa et al. (2015) assessed negative experiences associated with gender identity, including expectations of rejection based on gender identity and internalization of transphobia. However, as Shulman et al. (2017) described, the measure has several limitations, including difficulty with scoring because many items are not on the same scale and insensitivity to change. Perhaps most importantly, the measure was designed for adults and does not fully address the stressors unique to adolescence described in the literature. TNBA minority stressors can be expected to differ from those experienced by transgender and non-binary adults due to the developmental period of adolescence, which is marked by identity development (Branje et al., 2021), increased risk of mental health symptom onset (Dahl and Gunnar, 2009), and exposure to minority stressors in adolescence-specific contexts of school and family (Goldbach and Gibbs, 2017). The specificity of this developmental period has previously merited development of analogous measures of minority stress for sexual minority adolescents; namely, the Sexual Minority Adolescent Stress Inventory (SMASI; Schrager et al., 2018). Thus, the present study sought to develop a valid and reliable measure of minority stress for use with TNBA between 13 and 17 years old: the Transgender Adolescent Stress Survey–Minority Stress (TASS-MS).

## 2 Preliminary studies

### 2.1 Interviews with TNBA adolescents

A first step to the present inquiry was a life history calendar (Caspi et al., 1996) study funded by the National Institutes on Child Health and Human Development [grant number 1F31HD091981] to conduct interviews with 20 TNBA to explore adolescent minority stress, parent support, and adolescent gender-affirmation processes during adolescence. Participants in these 90-to 120-min interviews were aged 12–17 and had initiated puberty blockers or gender-affirming hormones or both during the 12 months prior to the interview. These interviews are described in more detail elsewhere (Dunlap et al., 2023); however, a brief explanation of the coding relevant to the present study is described here.

After data were collected and transcribed, three researchers employed axial coding strategies to independently code text statements describing gender minority stressors. Statements were coded into conceptual domains and then inspected for fit with *a priori* (deductive) stress domains identified through prior literature reviews (e.g., transphobic communication, discrimination experiences). Additionally, prepublication versions of the SMASI (Goldbach and Gibbs, 2017; Schrager et al., 2018) were reviewed for gender-specific stress statements not included in the final published SMASI, which had heavily relied on SMA participants but potentially represented gender minority stress statements. These statements were incorporated into the initial draft measure, representing 145 initial minority stress items that advanced to the Delphi panel and process.

### 2.2 Modified Delphi process

Six experts in minority stress and TNBA health served as expert panelists in an item selection process using a modified version of the RAND/UCLA Appropriateness Method (Fitch et al., 2001) or modified Delphi process (Schrager and Goldbach, 2017). Following a 60-min training session, expert panelists independently rated each proposed minority stress item for content validity and feasibility on a 9-point scale (1 = *low*, 9 = *high*). A high validity score meant there is adequate scientific evidence or professional consensus supporting the content of the item, in that respondents with higher scores on the item would be considered to have experienced higher levels of stress. A high feasibility score meant that adolescent respondents are likely to find the item readable and comprehensible and self-report evaluation of the stressor is likely to be reliable and accurate. After a 3-week first-round rating period, panelist ratings were scored for acceptance with agreement, rejection with agreement, or discrepancy among the reviewers that required further discussion of the item. After the first round of ratings, 32 minority stress items were accepted and the remaining 123 items were discrepant and required additional review by the panel.

Three expert panel meetings totaling 15 h were held via Zoom video conference. Of the 123 minority stress items to be discussed, 75 were retained for second-round ratings; the remaining 48 were rejected during the panel discussion. After the second round of expert panelist ratings, 67 items—including the 32 items previously accepted—were accepted as valid and feasible by the panel and included in cognitive interviews with TNBA.

### 2.3 Cognitive interviews

Fourteen TNBA (aged 12–17) were referred to participate in cognitive interviews via researcher contacts in local adolescent and family community-based programs. Youth were asked to report whether each proposed stress item was clear and understandable, offensive, difficult to understand, and realistic (“Is this something that you could see happening to somebody like you?”). Items that youth found to be unclear, offensive, or unrealistic were probed further, and youth were asked to recommend clearer or more appropriate language, which was then discussed by the study team. New and revised statements were also reviewed for content duplication with the SMASI to ensure item-level differentiation between the two measures. Finally, all items were copy-edited for consistency in verb tenses to ensure items reflected reportable lived experiences, in line with recommendations of Schrager and Goldbach (2017). Of the 67 items shared with youth participants, 44 items were retained without any conceptual changes, 22 items were revised based on youth feedback, one item was deleted, and three new items were added in response to participant recommendations. This resulted in a 69-item candidate gender minority stress measure that was advanced for inclusion in the main study.

## 3 Method

### 3.1 Participants and procedures

All methods were reviewed and approved by the institutional review board prior to beginning study activities. Participants (*N* = 444) in the main study were recruited and enrolled in two phases. Starting in January 2022, targeted advertisements were shown on social media platforms, specifically Instagram and YouTube; however, due to a change in Instagram advertising policies around this time, we could not target our audience from this platform using specific interest keywords. To narrow the scope of the recruitment campaign, advertisements were targeted by age, geographic region (West, Midwest, Southwest, Southeast, Northeast), and urbanicity using the 2020 Rural–Urban Commuting Area taxonomy for coding urbanicity by ZIP code (Cromartie, 2020). The advertisements promoted links to a subject pool screener, where youth entered their demographic and contact information and were informed that they would be contacted if they were eligible for future studies. Youth were eligible to participate in the present study if they were 12–17 years old; resided in the United States or a U.S. territory; responded “yes” to at least one of three questions assessing if they were transgender, non-binary, or genderqueer or if they did not identify with one of these three labels; reported any gender identity that did not match their sex assigned at birth; and were willing and able to provide assent to participate in the study.

After reviewing demographic information provided in the subject pool screener, the study team individually contacted eligible youth via the contact information they provided to invite them to participate in the initial study survey, which was an online survey deployed in Qualtrics. This survey initially asked demographic questions to verify study eligibility before advancing to the assent information screen. After providing assent, participants first completed a battery of 89 newly developed items, followed by validation measures. However, during pilot testing, the complete survey was determined to be too long and burdensome for adolescents. Thus, the validation measures were divided across three shorter survey versions. When invited to participate in the study, respondents were randomly assigned to receive one of the three survey versions, each containing the newly developed minority stress measure along with a subset of validation measures. In total, 444 participants completed one of the three primary survey versions between March and May 2022.

To ensure data quality, participants who completed the survey in an improbably short timespan or failed to complete at least three of four attention-control items correctly (e.g., “Please select ‘Decline to Answer’”) were excluded from the data prior to analysis (Bauermeister et al., 2012; Robinson-Cimpian, 2014). Participants who attempted to gain re-entry to the survey to receive additional compensation, such as via multiple attempts from the same IP address or providing contact information from an existing participant, were also excluded from analysis to prevent duplicate participation (Grey et al., 2015; Teitcher et al., 2015). Participants who successfully completed the baseline survey were invited to recruit additional participants from their personal networks in a respondent-driven sampling process (Heckathorn, 1997). Participants received an email containing three unique survey links and language prompts to encourage peers to participate. For each eligible participant who completed the baseline survey, the participant who referred that individual received an additional $10 online gift card.

**Figure 1:**
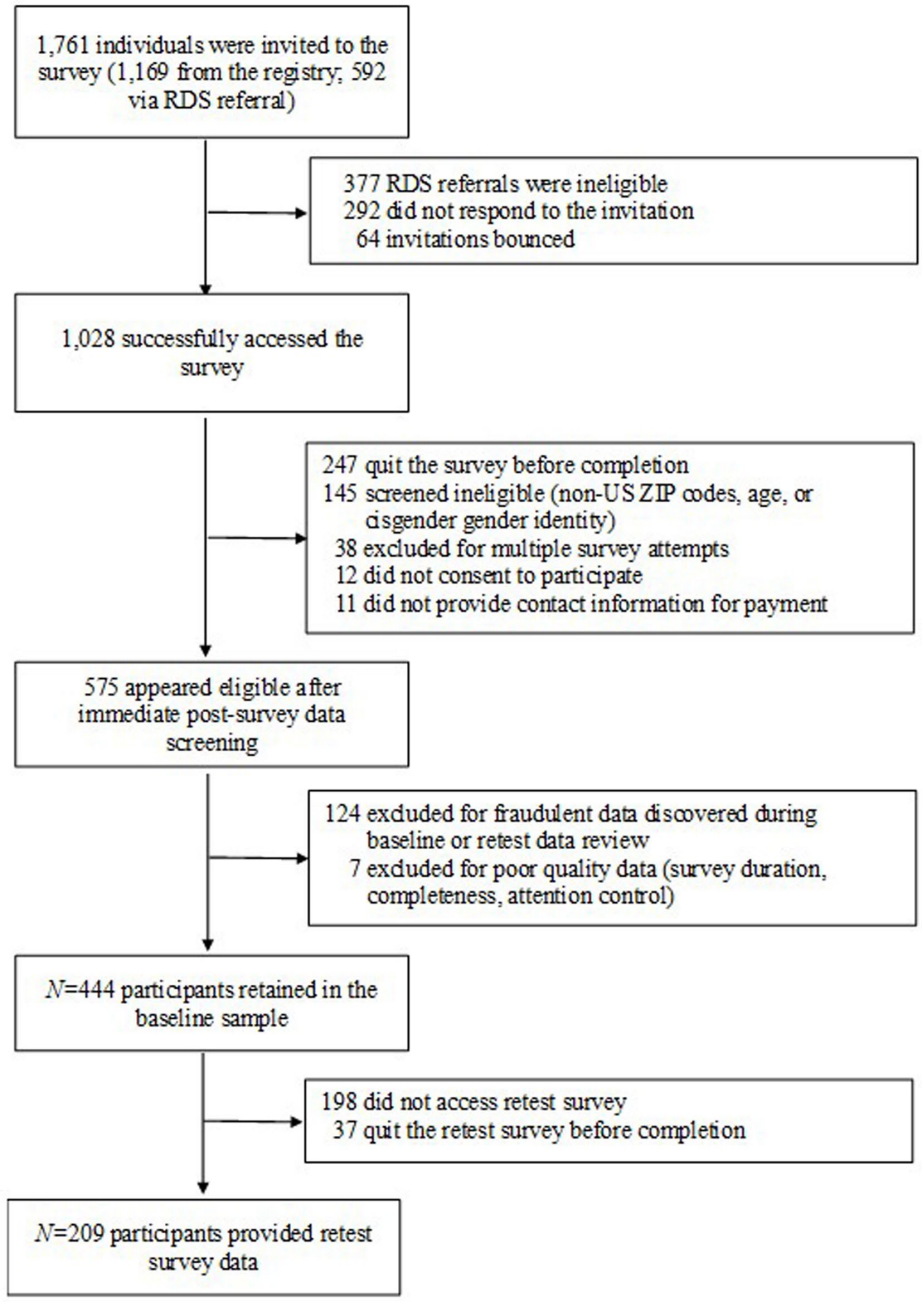
CONSORT flow diagram for study enrollment (final *N* = 444 for baseline, *N* = 209 for retest).

Two weeks after participants took the initial survey, they were contacted again with a request to complete a retest survey of only the newly developed measures and provide sufficient demographic information to verify accurate data file matching. Participants received a $25 online gift card for completion of the baseline survey and a $10 online gift card for completion of the retest. Of the 444 participants who completed the initial survey, 246 (55.4%) also participated in the retest survey, which was completed between April and June 2022 based on each respondent’s baseline participation date. The full CONSORT diagram illustrating participant recruitment and retention through the test–retest period is presented in Figure 1.

### 3.2 Measures

All participants completed the following measures:

#### 3.2.1 Gender-related minority stress

The key measure in the current study, the candidate minority stress measure, consisted of 69 statements describing gender-related minority stress experiences. For each statement, participants were asked to report whether they had experienced the stressor in their lifetime (1 = *yes*, 0 = *no*). For each item endorsed, participants were subsequently asked whether that experience had occurred in the past 30 days (1 = *yes*, 0 = *no*).

#### 3.2.2 Eligibility-related demographics

To verify eligibility to participate, participants were again asked to report their age; country of residence (recoded as 1 = *United States*, 0 = *all others*); five-digit ZIP code; sex assigned at birth (0 = *female*, 1 = *male*); binary indicators (1 = *yes*, 0 = *no*) of whether they identify as transgender, non-binary, or genderqueer; and self-reported gender identity (Table 1).

**Table 1:** Demographic descriptive statistics.

Variable	*n* (%)
Race and ethnicity	
White	291 (65.5)
Black	42 (9.5)
Hispanic or Latinx	42 (9.5)
Other	69 (15.5)
U.S. region	
Northeast	69 (15.5)
Southeast	100 (22.5)
Midwest	90 (20.3)
Southwest	57 (12.8)
West	123 (27.7)
Missing	5 (1.1)
Urbanicity	
Urban	367 (82.7)
Rural	72 (16.2)
Missing	5 (1.1)
Age group	
12–14	64 (14.4)
15	107 (24.1)
16	163 (36.7)
17	110 (24.8)
Pubertal development	
Minimal development, no blockers or hormones	82 (18.5)
Significant development, no blockers or hormones	252 (56.7)
Experience with blockers or hormones	83 (18.7)
Missing	27 (6.1)
Sex assigned at birth	
Female	327 (73.6)
Male	117 (26.4)
Sexual identity	
Gay/Lesbian	116 (26.1)
Bisexual/Pansexual	229 (51.6)
Asexual	56 (12.6)
Queer/Unlabeled/Other	43 (9.7)
Gender identity	
Transmasculine	154 (34.7)
Transfeminine	77 (17.3)
Nonbinary	170 (38.3)
Agender	42 (9.5)
Missing	1 (0.2)

#### 3.2.3 Other demographics

Although not used to determine eligibility, all participants were also asked to report their sexual identity or orientation; race and ethnicity; current school enrollment (binary); highest grade completed; a 4-point item assessing family socioeconomic status (“does not meet basic needs” to “live comfortably”); prior and current employment; current living situation; experiences of homelessness (assessed with one item, “Have you ever had to spend the night somewhere other than your home because you had nowhere else to stay?” with positive responses incurring follow-up items asking about experiences with different types of shelter); personal and family religion; binary items assessing prior and current use of pubertal blockers or hormone replacement therapy; and five items assessing pubertal development.

#### 3.2.4 Concurrent validity measures

Behavioral health outcomes used to establish concurrent validity included the Center for Epidemiological Studies Depression Scale-4 (CES-D-4; Melchior et al., 1993), an abbreviated version of the revised full version containing four self-report items that measure past-week depressive symptoms (*α* = 0.83). Anxiety symptoms were measured using the 7-item Generalized Anxiety Disorder-7 (GAD-7; Spitzer et al., 2006), which assesses how often a person has been bothered by certain problems during the past 2 weeks (*α* = 0.88). The 6-item PTSD Checklist – Civilian (PCL-C-6; Lang et al., 2012) is an abbreviated 6-item version of the 17-item version that assesses symptoms of PTSD (α = 0.82). Suicidality was assessed with five items from the Youth Risk Behavior Survey (Centers for Disease Control and Prevention, 2010) and recoded into binary indicators (1 = *yes*, 0 = *no*) of past 12-month suicidal ideation, suicide attempt, and non-suicidal self-injury. Finally, lifetime and past-30-day use of alcohol, marijuana, and other drugs were measured with the corresponding substance use items from the Youth Risk Behavior Survey. “Other drugs” included use of illicit drugs (e.g., cocaine, heroin, hallucinogens) and misuse of prescription drugs (opiates and stimulants).

#### 3.2.5 Convergent and divergent validity measures

During pilot testing of the full-length survey with the study team, the complete survey was determined to be too long and burdensome for adolescents. Thus, the validation measures were divided across three shorter survey versions. At the time they were invited to participate in the study, respondents were randomly assigned to receive one of the three survey versions, each of which contained a subset of validation measures, as follows.

#### 3.2.6 Survey A

The first survey version (*n* = 121) included two divergent validation measures: the 18-item Utrecht Gender Dysphoria Scale-Gender Spectrum (UGDS-GS; McGuire et al., 2020; *α* = 0.90) and the Gender Identity/Gender Dysphoria Questionnaire for Adolescents and Adults (GIDYQ-AA; Deogracias et al., 2007), which has a 27-item version intended for participants assigned female at birth (GIDYQ-FAB, *α* = 0.82) and a separate 27-item version for use with participants assigned male at birth (GIDYQ-MAB, α = 0.80). Scoring for these measures follows the scoring procedures in the original citations; higher GIDYQ-AA scores indicate lower levels of gender dysphoria.

#### 3.2.7 Survey B

The second version (*n* = 142) contained the 60-item GMSR (Hidalgo et al., 2019), used for convergent validation. The GMSR does not yield a single total score but rather nine subscale scores reflecting gender-related discrimination, rejection, and victimization; gender identity non-affirmation; internalized transphobia; negative expectations for the future; non-disclosure of gender identity or history; pride; and community connectedness (α = 0.69–0.89). Higher scores on the GMSR indicate higher agreement with statements corresponding to the construct of each subscale, except for gender-related discrimination, rejection, and victimization subscales, in which high scores indicate more lifetime experience of types of discrimination, rejection, and victimization, respectively.

#### 3.2.8 Survey C

The third and final survey version (*n* = 139) included the original Utrecht Gender Dysphoria Scale (UGDS; Cohen-Kettenis and van Goozen, 1997), which includes 12 items intended for participants assigned female at birth (UGDS-F, α = 0.88) and 12 different items for participants assigned male at birth, to assess divergent validity (UGDS-M, α = 0.88). Higher scores on the UGDS scales indicate higher levels of gender dysphoria. Survey C also included the SMASI (Goldbach et al., 2017; Schrager et al., 2018) to establish convergent validity. The SMASI includes 54 primary items assessing lifetime and 30-day LGBTQ minority stress, with an additional 10 items assessing work-related minority stressors for participants who were currently or previously employed (α = 0.92 for lifetime scores; α = 0.88 for past-30-day scores). Higher scores on the SMASI indicate experience of more types of sexual identity-related minority stressors.

### 3.3 TASS-MS analytical plan

To identify the structure of the TASS-MS, we fit a principal components analysis model to the lifetime versions of the 69 candidate items. Based on the number of components identified, we then fit candidate exploratory factor models to identify both the ideal number of factors (subscales) and which items loaded on which factors. Having identified an initial factor structure for the measure’s subscales, we then completed item response theory (IRT) and measurement invariance analyses. We fit IRT models to each candidate subscale to estimate the difficulty and discriminability of each item with respect to the latent construct measured by the subscale. Items with poor discriminability—poor differentiation between different levels of the latent trait—were dropped from subscales at this point. Then we proceeded to measurement invariance analyses across different levels of demographic variables, sequentially testing configural (fixed pattern of factor loadings) and metric (fixed item loadings) invariance models. We tested measurement invariance across race and ethnicity, U.S. region, urbanicity, age group, gender identity, sexual identity, sex assigned at birth, and pubertal development variables. Variables that contributed to measurement non-invariance, especially with respect to configural invariance and metric invariance, were dropped from subscales at this stage.

Having arrived at a final structure of the TASS-MS, we conducted reliability and validity analyses. Reliability was assessed with McDonald’s omega coefficient for dichotomous variables. Validity analyses examined the correlation between TASS-MS scores and existing measures for gender-related minority stress and other constructs, as well as the relationship between the TASS-MS and behavioral health variables hypothesized to be related to minority stress (i.e., depression, anxiety, PTSD, suicidal behavior, and substance use).

## 4 Results

### 4.1 TASS-MS structural analysis

Through exploratory factor analysis for categorical data using varimax rotation in Mplus, the eigenvalue decomposition of the covariance matrix indicated that up to four factors would be sufficient to explain the variability in TASS-MS scores, as judged by the inflection point in the scree plot of eigenvalues. Following this indication, we fit exploratory factor models of the initial 69 lifetime items with one to four factors in SPSS, using the principal components analysis extraction method for dichotomous data (Meulman et al., 2004). Items with factor loadings greater than or equal to 0.50 were retained for further analysis in confirming the structure of the TASS-MS subscales. At this stage, we initially identified a three-factor model as optimal to explain the data, based on model fit and parsimony. This model was composed of initial subscales made up of 14 items, 11 items, and seven items (initial items and full factor estimates for this model can be found in Supplementary Table S1). Thematic analyses of the items contained in these factors by experts on our team identified the factors as measuring minority stress related to disaffirmation, visibility and internalized transnegativity, and gender-related minority stress in the family context, respectively.

### 4.2 Item response theory

We fit a two-parameter Rasch IRT model for dichotomous data to each subscale. The Rasch model estimates two item response properties for each item: item difficulty (level of latent trait at which one has a 50% chance of endorsing an item) and item discriminability (how precisely the probability of endorsing an item corresponds to a particular value of the latent trait). Because the Rasch model assumes that the underlying latent construct is univariate, we fit IRT models separately to each candidate subscale. IRT models were fit using the *ltm* function from the *ltm* package in R (Rizopoulos, 2006).

#### 4.2.1 Disaffirmation subscale

**Figure 2:**
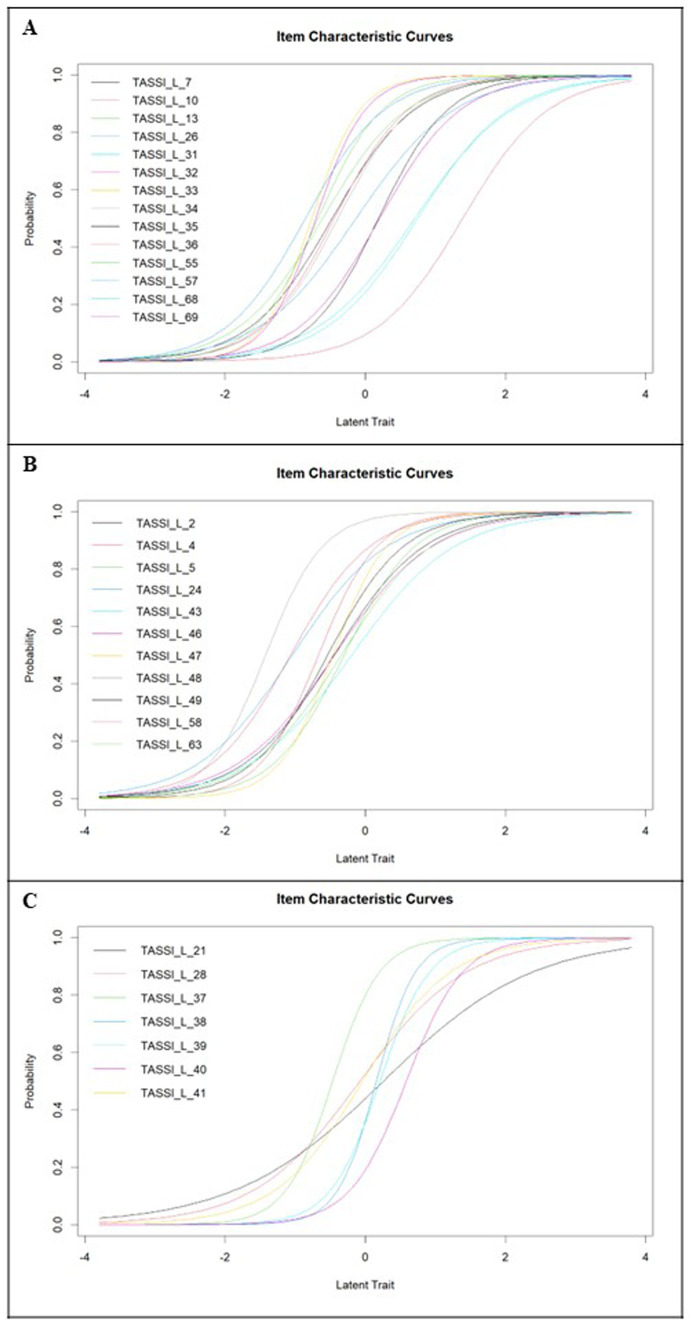
**(A)** Item characteristic curves for TASS-MS disaffirmation items; **(B)** item characteristic curves for TASS-MS visibility and internalized transnegativity items; and **(C)** item characteristic curves for TASS-MS family items.

This factor consisted of 14 items at outset. Item characteristic curves from the Rasch model are shown in Figure 2A, plotting the probability of endorsing an item along different values of a latent trait. Difficulty estimates ranged from-0.85 (Item 57) to 1.39 (Item 36). Discriminability estimates were all acceptable, ranging from 1.42 (Item 26) to 2.87 (Item 33). No items were excluded from this subscale based on poor IRT properties.

#### 4.2.2 Visibility and internalized transnegativity subscale

This subscale consisted of 11 items at outset. Item characteristic curves are shown in Figure 2B. Difficulty estimates ranged from-1.42 (Item 48) to-0.19 (Item 43), indicating that the items comprising this subscale were commonly endorsed among participants in our study. This does not, however, indicate that minority stress experienced from not being able to live or express one’s gender identity authentically is necessarily “mild” in terms of the latent construct. Discriminability estimates were all acceptable, ranging from 1.36 (Item 43) to 2.59 (Item 47).

#### 4.2.3 Family subscale

This scale consisted of seven items at outset. Item characteristic curves for the family subscale are shown in Figure 2C. Difficulty estimates ranged from-0.46 (Item 37) to 0.58 (Item 40). Item discriminability ranged from 0.94 (Item 21) to 3.59 (Item 38). The discriminability estimate for Item 21 was borderline acceptable, but we retained the item in the family subscale for the next stage of measurement invariance analyses.

### 4.3 Measurement invariance testing

Measurement invariance was conducted across race and ethnicity, urbanicity, U.S. region, age group, sex assigned at birth, pubertal development and experience with hormones or puberty blockers, sexual identity, and gender identity. Changes in the comparative fit index (CFI) between configural (same factor structure but no constraints on factor loadings or means between groups) and metric (constrained equal factor loadings and thresholds) models were used to determine whether the more constrained model showed a decrement in fit compared to the baseline configural model. ΔCFI >0.01 prompted further investigation of factor loadings and thresholds. Measurement invariance testing was carried out in MPlus version 8.9 (Muthén and Muthén, 1998-2017).

Initially, several items were found to contribute to measurement invariance. Item 48 (“I wish being transgender or non-binary wasn’t so hard”) was removed from the visibility and internalized transnegativity subscale to improve non-invariance across urbanicity and sex assigned at birth. Item 21 (“I was forced to present my gender differently when I was at a place of worship”) and Item 28 (“I had to sneak around family to access community resources that supported my gender”) were removed from the family subscale to address measurement invariance across sex assigned at birth and gender identity.

#### 4.3.1 Race and ethnicity

Race and ethnicity was characterized as White, Black, Hispanic or Latinx, and other. Metric invariance was supported for the disaffirmation and family subscales by all metrics with good localized fit (CFI = 0.925–0.970). The visibility and internalized transnegativity subscale showed a significant difference in fit between the configural and metric invariance models, Δχ^2^(59) = 79.76, *p* = 0.04, but the CFI indicated improved fit of the metric model (configural CFI = 0.938, metric CFI = 0.954), as did the root mean square error of approximation (RMSEA; configural RMSEA = 0.094, 90% CI [0.770, 0.110]; metric RMSEA = 0.068, 90% CI [0.052, 0.083]).

#### 4.3.2 Urbanicity

Metric invariance across urban and rural status was supported by all three metrics for the family and disaffirmation factors (CFI = 0.960–0.991, ΔCFI = 0.00–0.10). The visibility and internalized transnegativity subscale showed a significant difference in fit between the configural and metric invariance models, Δχ^2^(19) = 35,050, *p* = 0.01; however, the change in CFI was <0.01 (configural CFI = 0.952, metric CFI = 0.960) and CFI values indicated good fit. Changes in RMSEA were also small (configural RMSEA = 0.086, 90% CI [0.069, 0.103], metric RMSEA = 0.065, 90% CI [0.050, 0.079]).

#### 4.3.3 U.S. region

U.S. region was divided into five categories: Northeast, Southeast, Midwest, Southwest, and West. Metric invariance was supported by all metrics for the visibility and internalized transnegativity and family factors. The chi-square difference test detected a significant difference in fit between the configural and metric invariance on the disaffirmation factor, Δχ^2^(111) = 169.35, *p* < 0.01. However, the other fit metrics did not show a dramatic difference between these models (ΔCFI = 0.002; ΔRMSEA = 0.007).

#### 4.3.4 Age group

Participants were divided into age four groups for analysis: 12–14, 15, 16, and 17. Metric invariance was supported by all metrics for all three subscales. CFI additionally indicated good localized fit for each factor (CFI = 0.953–0.999).

#### 4.3.5 Sex assigned at birth

Sex assigned at birth was categorized as male or female. For the disaffirmation factor, CFI and RMSEA indicated good fit of the metric invariance model (CFI = 0.947, RMSEA = 0.082, 90% CI [0.072, 0.091]), comparable to that of the configural model (CFI = 0.947, RMSEA = 0.089, 90% CI [0.079, 0.099]). For the visibility and internalized transnegativity factor, CFI and RMSEA indicated improved fit of the metric invariance model (CFI = 0.936, RMSEA = 0.085, 90% CI [0.072, 0.098]) over the configural model (CFI = 0.914, RMSEA = 0.110, 90% CI [0.096, 0.125]). The metric invariance model was also supported for the family factor, with identical CFI for the configural and metric invariance models (CFI = 0.983) and similar RMSEA (configural RMSEA = 0.104, 90% CI [0.068, 0.142], metric RMSEA = 0.078, 90% CI [0.049, 0.108]).

#### 4.3.6 Self-reported pubertal development and experience with blockers or hormones

Pubertal development (self-reported) was combined with experience with puberty blockers or hormone replacement therapy to form three categories: minimal pubertal development with no experience with blockers or hormones, extensive pubertal development with no experience with blockers or hormones, and any stage of pubertal development with experience with blockers or hormones. Metric measurement invariance was supported for the family and visibility and internalized transnegativity factors. The disaffirmation factor showed a significant change in fit by the chi-square difference test, Δχ^2^(55) = 111.6, *p* < 0.001, but not by the changes in CFI (metric: 0.958, configural: 0.961) or RMSEA (configural: 0.079, 90% CI [0.068, 0.091]; metric: 0.074, 90% CI [0.063, 0.085]).

#### 4.3.7 Sexual identity

Sexual identity was divided into four categories: gay or lesbian, bisexual or pansexual, asexual, and queer. No factors indicated worse or significantly different fit of the metric invariance model when compared to the configural invariance model. Additionally, all factors indicated good localized fit of the metric invariance model (CFI = 0.969–0.993).

#### 4.3.8 Gender identity

Gender identity was divided into four categories: transfeminine, transmasculine, agender, and non-binary. Metric measurement invariance was supported by CFI, RMSEA, and the chi-square test of difference across the visibility and internalized transnegativity and family subscales. The chi-square test of difference between the configural and metric model showed a significant difference of fit for the disaffirmation subscale, Δχ^2^(76) = 103.62, *p* = 0.02, but no substantial change in fit by the other metrics (configural: CFI = 0.951, RMSEA = 0.077, 90% CI [0.065, 0.088]; metric: CFI = 0.954, RMSEA = 0.066, 90% CI [0.054, 0.077]).

### 4.4 TASS-MS scoring

The final TASS-MS consisted of three subscales: disaffirmation (14 items), visibility and internalized transnegativity (10 items), and family (five items), for a total of 29 items (full items can be found in Appendix A). Items were scored yes (1) or no (0), and subscale and total scores were calculated as the sum of binary-coded answers. If a participant was missing three or fewer items on the total scale, then we substituted the mean of their other answers (i.e., proportion endorsed) for the missing values. If a participant declined to answer more than three items, then the entire score was considered missing (*n* = 394 for past-30-day scores, *n* = 401 for lifetime scores). The mean of lifetime TASS-MS scores was 16.36 (*SD* = 7.07), and the mean of past-30-day scores was 10.42 (*SD* = 6.62).

### 4.5 Reliability

Reliability analyses, including versions with each item removed, were conducted to assess the internal consistency of the TASS-MS. Because the measure’s items are binary, we used a measure of composite reliability for categorical items using the *compRelSEM* function from the *semTools* package in R (Jorgensen et al., 2022). Reliability estimates for all three factors were acceptable (disaffirmation: *ω* = 0.943; visibility and internalized transnegativity: ω = 0.896; family: ω = 0.859). Omission of any item did not improve subscale reliability substantially, so all items were retained at this stage.

A subset of approximately half of participants took a retest of the TASS-MS items 2 weeks after initial assessment. Because of the time-sensitive nature of the past-30-day assessment, only the lifetime items were analyzed for test–retest reliability. For the lifetime TASS-MS, test–retest reliability was *r* = 0.81 (95% CI [0.75, 0.86]). Test–retest reliability of the lifetime disaffirmation subscale was *r* = 0.82 (95% CI [0.76, 0.86]). Test–retest reliability of the lifetime visibility and internalized transnegativity subscale was *r* = 0.76 (95% CI [0.69, 0.81]). Finally, test–retest reliability of the lifetime family subscale was *r* = 0.74 (95% CI [0.67, 0.80]).

### 4.6 Convergent and divergent validity

Table 2 shows the correlations between the TASS-MS total and subscale lifetime scores with several other measures of gender minority stress and gender dysphoria. We observed moderate correlations between the TASS-MS total and subscale scores and measures of gender dysphoria, including the UGDS-F, UGDS-GS, and GIDYQ-AA forms. Observed correlations between the TASS-MS family subscale scores and measures of gender dysphoria were weaker or non-significant.

**Table 2:** Correlations between TASS-MS lifetime scores and subscales with other measures of gender minority stress and dysphoria.

TASS-MS lifetime	Whole scale	Disaffirmation	Visibility and internalized transnegativity	Family
UGDS-F	0.45*	0.50*	0.26*	0.15
UGDS-M	0.07	0.26	0.01	−0.09
UGDS-GS	0.30*	0.23*	0.24*	0.17*
GIDYQ-FAB	−0.45*	−0.51*	−0.24*	−0.20*
GIDYQ-MAB	−0.37*	−0.34*	−0.34*	−0.05
SMASI Lifetime	0.66*	0.41*	0.60*	0.55*
GMSR Discrimination	0.33*	0.43*	0.09	0.14
GMSR Rejection	0.66*	0.59*	0.43*	0.45*
GMSR Victimization	0.51*	0.57*	0.21*	0.27*
GMSR Non-affirmation	0.39*	0.34*	0.23*	0.32*
GMSR Internalized transphobia	0.36*	0.16	0.39*	0.29*
GMSR Pride	−0.20*	−0.07	−0.26*	−0.01
GMSR Negative expectancies				
Form A	0.60*	0.41*	0.51*	0.45*
Form B	0.46*	0.37*	0.41*	0.34*
GMSR nondisclosure				
Form A	0.39*	0.24	0.26	0.37*
Form B	0.53*	0.48*	0.45*	0.30*
GMSR community connectedness	0.06	0.11	−0.01	0.03

UGDS, Utrecht Gender Dysphoria Scale; GIDYQ, Gender Identity/Gender Dysphoria Questionnaire; SMASI, Sexual Minority Adolescent Stress Inventory; GMSR, Gender Minority Stress and Resilience Measure; FAB, female at birth; MAB, male at birth; GS, gender spectrum. Parameters significant at least at p < 0.05 are indicated by *..

In contrast, the GMSR captures various constructs related to gender minority stress and resilience. The majority of GMSR minority stress subscales were positively and moderately (*r* = 0.30–0.60) correlated with the TASS-MS lifetime total scores and subscale scores. Community connectedness from the GMSR was not correlated with any TASS-MS scores, and the GMSR pride subscale was weakly negatively correlated with TASS-MS total scores and the visibility and internalized transnegativity subscale, but not the disaffirmation or family subscales. Correlations of the TASS-MS and subscales with lifetime SMASI scores were moderate and positive (*r* = 0.66, *p* < 0.001 for the TASS-MS; subscale correlations ranged from 0.41–0.60).

### 4.7 Concurrent validity

To assess concurrent validity, we assessed the associations between the TASS-MS and its subscales with outcomes assessing mental health, suicidal ideation and behavior, and substance use. Multiple regression, controlling for demographic variables, was used for continuous outcomes, and logistic regression, also controlling for demographic variables, was used for binary outcomes. Tables 3, 4 give the standardized regression estimates and odds ratios associated with TASS-MS total and subscale scores for the lifetime and past-30-day scores, respectively.

**Table 3:** Standardized regression estimates and odds ratios associated with the TASS-MS and subscale lifetime scores.

Outcome	TASS-MS lifetime	Disaffirmation subscale	Visibility and internalized transnegativity subscale	Family subscale
Continuous outcomes				
Depression (CES-D-4)	0.32*	0.20*	0.32*	0.22*
Anxiety (GAD-7)	0.35*	0.27*	0.32*	0.21*
PTSD (PCL-C-6)	0.33*	0.26*	0.26*	0.28*
Binary outcomes				
Suicidal ideation	1.14*	1.19*	1.30*	1.34*
Suicide attempt	1.12*	1.21*	1.21*	1.28*
Non-suicidal self-injury	1.09*	1.14*	1.15*	1.26*
Alcohol use				
Past 30 days	0.98	0.96	0.92	1.06
Lifetime	1.00	1.00	0.97	1.02
Tobacco use				
Past 30 days	0.95*	0.92*	0.90*	0.94
Lifetime	0.99	0.98	0.95	1.04
Marijuana use				
Past 30 days	1.08*	1.15*	1.11*	1.21*
Lifetime	1.08*	1.17*	1.10*	1.13
Prescription drug use				
Past 30 days	1.10*	1.25*	1.12	1.08
Lifetime	1.01	1.00	0.96	1.22*
Illicit drug use				
Past 30 days	NA	NA	NA	NA
Lifetime	1.03	0.97	1.07	1.35

Parameter estimates associated with the TASS-D and its subscales are taken from multiple regression (for continuous outcomes) and logistic regressions (for binary outcomes) that control for demographic features. Standardized regression parameters are reported for continuous outcomes, odds ratios are reported for binary outcomes. Outcomes where the estimate is reported as NA had insufficient occurrence in the data to estimate the model. Parameters significant at least at p < 0.05 are indicated by *.

**Table 4:** Standardized regression estimates and odds ratios associated with the TASS-MS and subscale past 30-day scores.

Outcome	TASS-MS 30-Day	Disaffirmation subscale	Visibility and internalized transnegativity subscale	Family subscale
Continuous outcomes				
Depression (CES-D-4)	0.38*	0.26*	0.34*	0.22*
Anxiety (GAD-7)	0.43*	0.30*	0.37*	0.30*
PTSD (PCL-C-6)	0.42*	0.31*	0.33*	0.34*
Binary outcomes				
Suicidal ideation	1.13*	1.19*	1.24*	1.31*
Suicide attempt	1.14*	1.22*	1.24*	1.32*
Non-suicidal self-injury	1.10*	1.16*	1.17*	1.27*
Alcohol use				
Past 30 days	0.97	0.99	0.91	1.01
Tobacco use				
Past 30 days	0.95*	0.92*	0.90	0.93
Marijuana use				
Past 30 days	1.03	1.10*	1.00	1.04
Prescription drug use				
Past 30 days	1.13*	1.31*	1.06	1.32
Illicit drug use				
Past 30 days	NA	NA	NA	NA

Parameter estimates associated with the TASS-D and its subscales are taken from multiple regression (for continuous outcomes) and logistic regressions (for binary outcomes) that control for demographic features. Standardized regression parameters are reported for continuous outcomes, odds ratios are reported for binary outcomes. Parameters significant at least at p < 0.05 are indicated by *. Outcomes where the estimate is reported as NA had insufficient occurrence in the data to estimate the model.

#### 4.7.1 Mental health outcomes

Lifetime and past-30-day total TASS-MS scores were associated with significantly higher scores for depression (lifetime: *β* = 0.32, *p* < 0.001; past-30-day: *β* = 0.38, *p* < 0.001), anxiety (lifetime: *β* = 0.35, *p* < 0.001; past-30-day: *β* = 0.43, *p* < 0.001), and PTSD (lifetime: *β* = 0.33, *p* < 0.001; past-30-day: *β* = 0.42, *p* < 0.001). The same patterns held true for all subscales of lifetime and past-30-day TASS-MS.

#### 4.7.2 Suicidal ideation and behavior

TASS-MS total scores were associated with higher odds of suicidal ideation for lifetime (*OR* = 1.14, 95% CI [1.10, 1.19]) and past-30-day scores (*OR* = 1.13, 95% CI [1.08, 1.18]). All three subscales, for both lifetime and past-30-day scores, were associated with significantly higher odds of suicidal ideation. Odds of suicide attempt were also significantly higher for those with higher TASS-MS lifetime total scores (*OR* = 1.12, 95% CI [1.07, 1.18]) and past-30-day total scores (*OR* = 1.14, 95% CI [1.08, 1.20]). All three subscales, in lifetime and past-30-day form, were associated with significantly higher odds of suicide attempt. Finally, lifetime TASS-MS total scores and past-30-day total scores were associated with significantly higher odds of non-suicidal self-injury (lifetime: *OR* = 1.09, 95% CI [1.05, 1.13]; past-30-day: *OR* = 1.10, 95% CI [1.06, 1.15]). Lifetime and past-30-day subscale scores were also associated with significantly higher odds of non-suicidal self-injury. Across all three outcomes, odds were particularly high for the visibility and internalized transnegativity and family subscales, compared to the odds associated with the total score.

#### 4.7.3 Substance use

##### 4.7.3.1 Alcohol

Neither TASS-MS lifetime nor past-30-day total scores were significantly associated with the odds of lifetime or past-30-day alcohol use. None of the TASS-MS subscales was significantly associated with the odds of either form of alcohol use.

##### 4.7.3.2 Tobacco

TASS-MS lifetime scores and lifetime disaffirmation scores and visibility and internalized transnegativity lifetime scores were significantly associated with slightly lower odds of past-30-day tobacco use (total score *OR* = 0.95, 95% CI [0.91, 0.99]). None of the lifetime scores was associated with the odds of lifetime tobacco use. Past-30-day TASS-MS total scores (*OR* = 0.95, 95% CI [0.90, 0.99]) and past-30-day disaffirmation subscale scores (*OR* = 0.92, 95% CI [0.84, 0.99]) were associated with slightly lower odds of past-30-day tobacco use, but the other past-30-day subscale scores were not.

##### 4.7.3.3 Marijuana

TASS-MS lifetime total scores were associated with higher odds of past-30-day marijuana use (*OR* = 1.08, 95% CI [1.04, 1.13]) and lifetime marijuana use (*OR* = 1.08, 95% CI [1.04, 1.12]). All lifetime subscales were also associated with higher odds of past-30-day marijuana use, and all but the lifetime family subscale were associated with higher odds of lifetime use. Of the past-30-day TASS-MS scores, only the disaffirmation subscale was significantly associated with the odds of past-30-day marijuana use (*OR* = 1.10, 95% CI [1.01, 1.20]).

##### 4.7.3.4 Prescription drugs

Prescription drug use included the use of prescription stimulants, pain killers, and tranquilizers. Lifetime TASS-MS total scores and disaffirmation scores were associated with significantly higher odds of past-30-day prescription drug use (lifetime total score *OR* = 1.10, 95% CI [1.00, 1.21]), but not odds of lifetime use. Only the lifetime family subscale was associated with significantly higher odds of lifetime prescription drug use (*OR* = 1.22, 95% CI [1.03, 1.46]). Past-30-day TASS-MS total scores and disaffirmation scores were also significantly associated with increased odds of past-30-day prescription drug use (past 30-day total score *OR* = 1.13, 95% CI [1.02, 1.26]).

##### 4.7.3.5 Illicit drugs

Illicit drugs included use of heroin, fentanyl, methamphetamine, and cocaine. Use of these drugs in our sample was very low, with only one occurrence of past-30-day use, such that models could not be fit for this outcome. TASS-MS total lifetime scores were not significantly associated with the odds of lifetime illicit drug use, nor were any lifetime subscale scores.

## 5 Discussion

This study sought to develop a novel measure of gender minority stress to understand the unique stressors that shape and contribute to health disparities among TNBA. Candidate items for the measure were composed based on prior life history interviews with TNBA; these candidate items underwent multiple review processes, including the RAND-UCLA expert panel Delphi process, cognitive interviews for item acceptability and comprehension with TNBA, and statistical analysis for factor analysis and invariance testing. The resulting measure of adolescent gender minority stress, the TASS-MS, is psychometrically sound, theoretically specific to the construct of gender minority stress, and developmentally appropriate for use with adolescents. Notably, this novel measure focuses on items specific to experiences of gender-related minority stress, such as pronoun use, ability to access restrooms, and parental gatekeeping to medical care, that are distinct for this population and separate from the types of stressors captured in measures intended for broader LGBT populations, such as the SMASI. This study provides a new option for measurement in accordance with changes in the theory and research on gender minority stress in adolescence and the need for measures inclusive of the experiences of non-binary adolescents.

As hypothesized and in accordance with the literature on minority stress and health, the TASS-MS and its subscales were all significantly associated with anxiety symptoms, depressive symptoms, PTSD symptoms, suicide ideation, suicide attempt, and non-suicidal self-injury. Although some hypothesized associations were found between TASS-MS and substance use, including marijuana and prescription drug use, we found no associations with alcohol use and negative associations with tobacco use. Future research may investigate whether these differences in substance use reflect actual relationships between minority stress and alcohol and tobacco use, developmentally specific substance use onset, or other unexamined factors such as access to various online and in-person social networks. Finally, the TASS-MS was moderately and weakly correlated with convergent and divergent measures, respectively, indicating that this novel measure is specific to the construct of minority stress.

Existing measures of gender-related minority stress and dysphoria have been criticized for inadequate construct differentiation (Shulman et al., 2017). The TASS-MS specifically assesses lifetime and recent gender minority stressors, to the exclusion of sexual minority stressors and gender dysphoria. The TASS-MS is unique in its approach to focusing on minority stressors that shape TNBA well-being while distinguishing between gender minority stress and body-related gender incongruence and dysphoria. Gender dysphoria has been recently argued to be a proximal minority stressor (Lindley and Galupo, 2020). Items that reflect incongruence between an adolescent’s sense of their gender and their body and that do not emphasize roots in exposure to a primary distal stressor (such as being misgendered) were parsed into a separate novel measure (Transgender Adolescent Stress Survey–Dysphoria; TASS-D) produced contemporaneously with this same measurement development process [blinded for review]. The decision to produce two measures, the TASS-MS and TASS-D, will ultimately allow future research to assess construct specificity and overlap. The TASS-MS conformity to the minority stress framework, to the exclusion of dysphoria-related items, resulted in a theoretically specific and valid measure of gender minority stress that can be paired with other theoretically driven measures such as the TASS-D and SMASI (Goldbach et al., 2017; Schrager et al., 2018).

Items in the TASS-MS reflect the experiences shared by TNBA in life history interviews, clinical and research expertise of Delphi panel members, and responses from TNBA in cognitive interviews. Whereas the TNBA life history interviews provided discrete experiences in a sample of TNBA adolescents upon which to base candidate items for the measure, the Delphi panelists and cognitive interviewees reviewed and edited each item for salience to TNBA life experiences. Delphi panelists and cognitive interviewees ensured that items would be comprehensible for adolescents. Items were edited, split, combined, or removed when interviewees struggled to understand them or, in some cases, indicated that they could be interpreted in multiple ways. Items were designed based on the experiences of binary and non-binary transgender adolescents and in some cases, items were dropped if cognitive interviewees and expert panelists indicated that the item would be answerable by only specific gender identities, to the exclusion of others. The resulting minority stress items, therefore, are answerable by youth of all ages and developmentally specific to adolescence, as evident in two subscales being specific to adolescent contexts of school and family. Measurement invariance testing generally supported the usability of this measure with youth across race and ethnicity, urbanicity, region, age, sex assigned, pubertal development and hormone or blocker use, sexual identity, and gender identity. Thus, the scale measures minority stress experiences that are understood by TNBA and provides researchers with opportunities to learn how different contexts, subgroup membership, development, and clinical interventions might differentially shape stress exposure throughout adolescence.

The study is not without limitations that should inform a cautious interpretation and use of study results. Original candidate items were composed based on life history interviews with TNBA who were receiving or about to receive either blockers or hormones. That study sample may have meaningfully differed from TNBA with reduced access to care (e.g., due to family, insurance, geography, sociopolitical contexts) or TNBA who do not desire medical intervention. However, the Delphi expert panel process, cognitive interviews, and subsequent validation of the items with a non-clinical sample of respondents largely alleviate this concern. Similar to a recent study with a nationwide sample of transgender adolescents (Salk et al., 2020), much of the study sample was assigned female at birth (73.6%), non-Hispanic White (65.5%), and living in an urban area (82.7%). Although we had sufficient statistical power to assess for invariance across these demographic groups, further research may seek more highly diverse samples to further understand within-group differences.

This study developed and tested a novel measure of gender minority stress for use with adolescents. The resulting measure is statistically valid, developmentally specific and appropriate, and inclusive of non-binary experiences. Minority stress research provides an etiological argument for why sexual minority people experience more mental health, substance use, and physical health challenges (Goldbach et al., 2021; Meyer, 2003; Testa et al., 2015). This research was instrumental in showing how social conditions shape health and combating arguments that sexual minority people are intrinsically unwell. This novel measure of gender minority stress offers a similar promise to furthering research that challenges essentializing narratives about TNBA and details the contexts and experiences that shape their well-being. It provides distinct benefits over existing measures, because it is inclusive of and usable by all gender minority adolescents, relies on a standard and simple scoring system, and assesses stressors specific to adolescence. The TASS-MS provides opportunities to examine, as has been done with SMA, how gender minority stress shifts in response to family, school, and policy interventions and historical change over time; etiological relationships between gender minority stress and health outcomes; and effects of mental health and physical health care on coping with minority stress.

## Data Availability

The raw data supporting the conclusions of this article will be made available by the authors, without undue reservation.

## Appendix A. Transgender adolescent stress scale - minority stress (TASS-MS)

**Table:**

TASS-MS: disaffirmation subscale (14 items)	
Original item #	Item text
7	People told me that I’m not really transgender or non-binary.
10	People made fun of my transgender or non-binary identity.
13	People told me that they cannot use my pronouns.
26	People seemed uncomfortable being around me because I am transgender or non-binary.
31	Problems accessing bathrooms or locker rooms caused me to be late for class.
32	I’ve felt uncomfortable using the bathroom at my school because I am transgender or non-binary.
33	I’ve felt uncomfortable using public bathrooms because I am transgender or non-binary.
34	I’ve felt uncomfortable using the locker room at my school because I am transgender or non-binary.
35	I was not allowed to use the locker room at school that matched my gender.
36	My peers harassed me about my gender when I used the locker room at school to change.
55	Teachers kept using the wrong pronouns for me.
57	People who knew my pronouns repeatedly misgendered me.
68	My participation in school sports was limited because I am transgender or non-binary.
69	School staff were not supportive of my gender identity.

**Table:**

TASS-MS: visibility and internalized transnegativity subscale (10 items)	
Original item #	Item text
2	I’ve been afraid to date because I am transgender or non-binary.
4	I’ve been afraid of others finding out that I am transgender or non-binary.
5	I’ve been afraid of being recognized as transgender or non-binary.
24	I had to wear clothes that do not reflect my gender.
43	I could not imagine a future where I would be accepted as my gender.
46	I worried that people would not date me because I am transgender or non-binary.
47	I’ve felt embarrassed about being transgender or non-binary.
49	I thought something was wrong with me because I am transgender or non-binary.
58	I believe people will treat me differently when they find out my gender history.
63	I have not felt safe expressing my gender at school.

**Table:**

TASS-MS: family subscale (5 items)	
Original item #	Item text
37	My parents did not help me live as my gender.
38	My parents tried to stop me from being transgender or non-binary.
39	My parents did not approve of me having transgender and non-binary friends.
40	My parents forbade me from presenting my gender in public.
41	A family member told me to present as my assigned sex at birth to certain people.
