# Supplementary material for: Development and validation of the Transgender Adolescent Stress Survey–Minority Stress (TASS-MS)
Source: Front Psychol. 2024 Dec 4;15:1448693. doi: 10.3389/fpsyg.2024.1448693 (PMC11653417; doi:10.3389/fpsyg.2024.1448693)
Supplement: Supplementary file 1 [file Table_1.docx]

**Supplementary Table 1. TASS-MS Item Bank and Factor Estimates for Three-Factor EFA**

| TASS-MS Item | Factor 1 | Factor 2 | Factor 3 |
| --- | --- | --- | --- |
| I’ve been afraid to disclose that I am transgender or nonbinary to someone I am dating. | .45 | .08 | .16 |
| I’ve been afraid to date because I am transgender or nonbinary. | .72 | -.11 | .19 |
| Someone threatened to out me as transgender or nonbinary. | -.06 | .26 | .51 |
| I’ve been afraid of others finding out that I am transgender or nonbinary. | .82 | .27 | .00 |
| I’ve been afraid of being recognized as transgender or nonbinary. | .74 | .10 | .08 |
| I’ve wanted to live my life without people knowing I am transgender or nonbinary. | .61 | -.03 | .06 |
| I’ve been afraid to disclose that I am transgender or nonbinary to someone I am dating. | .26 | .34 | .67 |
| I’ve been afraid to date because I am transgender or nonbinary. | .22 | .23 | .51 |
| Someone threatened to out me as transgender or nonbinary. | .26 | .09 | .49 |
| I’ve been afraid of others finding out that I am transgender or nonbinary. | .19 | .20 | .73 |
| I’ve been afraid of being recognized as transgender or nonbinary. | .00 | .44 | .46 |
| I’ve wanted to live my life without people knowing I am transgender or nonbinary. | .24 | .35 | .61 |
| People told me that I’m not really transgender or nonbinary. | .26 | .25 | .63 |
| People told me that they do not understand my gender. | .29 | .14 | .54 |
| A teacher made transphobic comments. | .44 | .11 | .08 |
| People made fun of my transgender or nonbinary identity. | .48 | .11 | .33 |
| My parents said that nobody will want to date me because I am transgender or nonbinary. | .29 | .09 | .03 |
| People said that nobody will want to be with me because I am transgender or nonbinary. | .34 | .08 | .25 |
| People told me that I’m not really transgender or nonbinary. | .65 | .40 | .05 |
| People told me that they do not understand my gender. | .17 | .40 | .63 |
| A teacher made transphobic comments. | .15 | .53 | .02 |
| People made fun of my transgender or nonbinary identity. | -.30 | .40 | .33 |
| My parents said that nobody will want to date me because I am transgender or nonbinary. | -.32 | .39 | .26 |
| People said that nobody will want to be with me because I am transgender or nonbinary. | .58 | .41 | .29 |
| People told me that they can’t use my pronouns. | .47 | .13 | .30 |
| I experienced transphobia from cisgender Lesbian, Gay, Bisexual (LGB) people. | .26 | .26 | .63 |
| There were no transgender or nonbinary role models like me in my life. | .26 | .20 | .52 |
| I’ve felt isolated at school because I am transgender or nonbinary. | .23 | .56 | .19 |
| I had to learn about my gender entirely alone. | .41 | .40 | .19 |
| I didn’t know any other transgender or nonbinary students at my school. | .34 | .13 | .68 |
| Has this happened to you within the past 30 days? People told me that they can’t use my pronouns. | .02 | -.14 | .69 |
| I experienced transphobia from cisgender Lesbian, Gay, Bisexual (LGB) people. | .24 | -.30 | .85 |
| There were no transgender or nonbinary role models like me in my life. | .24 | -.26 | .85 |
| I’ve felt isolated at school because I am transgender or nonbinary. | .25 | -.15 | .65 |
| I had to learn about my gender entirely alone. | .22 | .09 | .74 |
| I didn’t know any other transgender or nonbinary students at my school. | .10 | .08 | .70 |
| I had to hide my transgender or nonbinary identity. | .37 | .78 | .08 |
| People argued with me about how real my gender is. | .20 | .79 | .29 |
| I was forced to present my gender differently when I was at a place of worship. | .15 | .82 | .12 |
| I was not allowed to participate in religious ceremonies because I am transgender or nonbinary. | .01 | .81 | .11 |
| A religious leader tried to change my gender identity. | .35 | .59 | .47 |
| I had to wear clothes that do not reflect my gender. | .49 | .56 | .26 |
| I had to hide my transgender or nonbinary identity. | .61 | .15 | .20 |
| People argued with me about how real my gender is. | .44 | .04 | .47 |
| I was forced to present my gender differently when I was at a place of worship. | .76 | -.08 | .25 |
| I was not allowed to participate in religious ceremonies because I am transgender or nonbinary. | .81 | -.21 | .18 |
| A religious leader tried to change my gender identity. | .74 | .12 | .30 |
| I had to wear clothes that do not reflect my gender. | .79 | .11 | .43 |
| I had a hard time finding clothing that fits me and reflected my gender expression. | .62 | .23 | .39 |
| People seemed uncomfortable being around me because I am transgender or nonbinary. | .59 | .10 | .27 |
| People made assumptions about my sexual orientation based on my gender. | .50 | .21 | .27 |
| I had to sneak around family to access community resources that supported my gender. | .53 | .21 | .15 |
| I did not have access to gender affirming care. | .11 | .18 | .65 |
| I was not allowed to use a bathroom at school that matched my gender. | .18 | .23 | .59 |
| I had a hard time finding clothing that fits me and reflected my gender expression. | .43 | .18 | .70 |
| People seemed uncomfortable being around me because I am transgender or nonbinary. | .22 | .12 | .61 |
| People made assumptions about my sexual orientation based on my gender. | .22 | .08 | .66 |
| I had to sneak around family to access community resources that supported my gender. | .62 | .17 | .31 |
| I did not have access to gender affirming care. | .06 | .19 | .55 |
| I was not allowed to use a bathroom at school that matched my gender. | .06 | .15 | .59 |
| Problems accessing bathrooms or locker rooms caused me to be late for class. | .31 | .32 | .46 |
| I’ve felt uncomfortable using the bathroom at my school because I am transgender or nonbinary. | .50 | .57 | -.02 |
| I’ve felt uncomfortable using public bathrooms because I am transgender or nonbinary. | .66 | .28 | .22 |
| I’ve felt uncomfortable using the locker room at my school because I am transgender or nonbinary. | .54 | .27 | .27 |
| I was not allowed to use the locker room at school that matched my gender. | .56 | .24 | .18 |
| My peers harassed me about my gender when I used the locker room at school to change. | -.27 | .19 | -.01 |
| Problems accessing bathrooms or locker rooms caused me to be late for class. | .55 | .09 | .36 |
| I’ve felt uncomfortable using the bathroom at my school because I am transgender or nonbinary. | .13 | .00 | .65 |
| I’ve felt uncomfortable using public bathrooms because I am transgender or nonbinary. | .28 | .28 | .68 |
